# Nutritional Profile of the Ethiopian Oilseed Crop Noug (*Guizotia abyssinica* Cass.): Opportunities for Its Improvement as a Source for Human Nutrition

**DOI:** 10.3390/foods10081778

**Published:** 2021-07-31

**Authors:** Sewalem Tsehay, Rodomiro Ortiz, Mulatu Geleta, Endashaw Bekele, Kassahun Tesfaye, Eva Johansson

**Affiliations:** 1 Department of Plant Breeding, Swedish University of Agricultural Sciences, P.O. Box 190, SE-23422 Lomma, Sweden; sewalem.tsehay@slu.se (S.T.); rodomiro.ortiz@slu.se (R.O.); Eva.Johansson@slu.se (E.J.); 2 Department of Microbial, Cellular and Molecular Biology, Addis Ababa University, Addis Ababa P.O. Box 1176, Ethiopia; endashawbw@gmail.com (E.B.); kassahuntesfaye@yahoo.com (K.T.)

**Keywords:** *Guizotia abyssinica*, lipids, mineral elements, noug, oilseeds, protein

## Abstract

The aim of this study was to evaluate the potential of noug as a source for human nutrition. Diverse noug genotypes were evaluated for their content and/or composition of total lipids, fatty acids, proteins, and minerals using standard methods. The total lipid content (32.5–45.7%) and the proportion of an essential fatty acid, linoleic acid (72.2–77.8%), were high in noug, compared to other oilseed crops. The proportion of oleic acid, a monounsaturated fatty acid, was low in noug (5.2–9.2%). The breeding objective of increasing the oleic acid level in the highland, where noug is mainly cultivated, was limited, as the content of this acid was low in this environment. The seed protein concentration (25.4–27.5%) and mineral content were mainly affected by the cultivation environment, as the high temperature increased the amount of protein, whereas the soil condition was a major factor in the variation of the mineral content. Thus, noug is a unique crop with a high seed oil content, of which a high proportion is linoleic acid. With the exception of the seed oleic acid content, when grown in low-altitude areas, the genotypic variation contributes less than the cultivation environment to the nutritional attributes of noug. Hence, high-oleic-acid noug for lowland production can be targeted as a breeding goal.

## 1. Introduction

Oilseeds have the potential to contribute a vital source of nutrition to the human diet [1]. Vegetable oils, proteins, and minerals are components present in oilseeds, all having essential functions for the human body [1,2]. The profile of the content and composition of nutrients in a specific oilseed vary based on the species, type, maturity, environmental factors, breeding objectives, and management [3,4].

Currently, oilseeds are primarily used for oil extraction, and they are considered as the main sources of vegetable oil [5]. The quality of the seed oil is mainly determined by its fatty acid composition [6], which is known to have an impact on human health; e.g., soybean, corn, nuts, sunflower, safflower, and noug seed oils are rich in omega-6 fatty acids, which play a role in preventing cardiovascular disorders [7]. The oil extraction also results in a side product in the form of a press-cake, which is mainly used as animal feed, where its nutritional composition plays a role [8,9]. However, traditionally, oilseeds are consumed as a wholegrain food component. For example, in Ethiopia, slightly roasted noug (*Guizotia abyssinica* Cass.) seeds are finely pounded using a mortar and pestle, until a thick fluid, locally known as *litlit*, is formed. Then, bread made from different cereals, sugar, salt, etc., are added to the *litlit* and further pounded. The mixture is then made into balls of different sizes and served as a food locally known as *chifko* [10]. The current growing global population calls for an increased use of whole-seed oilseeds and/or oilseed cakes as a human nutrient source due to their high nutritive profile [11,12]. The proteins from oilseeds are known to have a high content of essential amino acids and are therefore beneficial to human health and well-being [13]. The oilseed minerals [14] have the potential to play an essential role in the human body by providing both macro- and micronutrients [15]. Mineral intake in sufficient amounts is essential for a vital life in humans and animals, and the consumption of inadequate amounts may result in an inefficient structure of muscles and the malfunction of nerves and metabolic processes, contributing to, e.g., threatened immunity, cognitive memory, and regulatory functions [16,17].

Noug is one of the major sources of edible seed oil in Ethiopia [18,19]. The vegetable seed oil from noug contributes significant economic and nutritional values to the country and its population, and it is ranked as a superior seed oil among Ethiopians [20]. Noug seeds have a relatively high trade value, contributing income to Ethiopia through its export [21]. A total lipid content of 25–56% has been shown for noug grown in Ethiopia [22,23,24,25,26,27]. The main fatty acids in noug oil are linoleic acid (C18:2), oleic acid (C18:1), palmitic acid (C16:0), and stearic acid (C18:0), contributing more than 90% of the total fatty acids in the seed oil [27]. The protein content of whole-seed noug has reported been limited, although seed meal of Ethiopian noug has been reported to contain about 30% protein [28]. A proximate analysis of noug seeds grown in the USA showed a higher level of protein content (28.2%) than imported noug seeds from Ethiopia (18.3%) and India (26.6%) [19]. A few studies have also evaluated the content of macro- and microminerals in the noug seed, and variations of these elements have been reported [29,30,31]. While several studies have been conducted on noug seed oil content and fatty acid composition, there is a lack of broad investigations covering the full nutritional potential of noug.

Hence, the objective of this paper was to investigate the quantity and quality of the nutrients of noug seeds, including lipids, fatty acids, proteins, and minerals, sampled from a broad range of materials, including cultivars, landraces collected from wide geographic areas in Ethiopia, and “breeding populations”, selected based on lipid content and yield parameters. The nutritive value of noug and opportunities for production of high-nutritive-value noug in Ethiopia were evaluated, and comparisons of the lipid and fatty acid composition with other major oilseed crops were carried out.

## 2. Materials and Methods

### 2.1. Plant Material and Field Experiments

**Figure 1:**
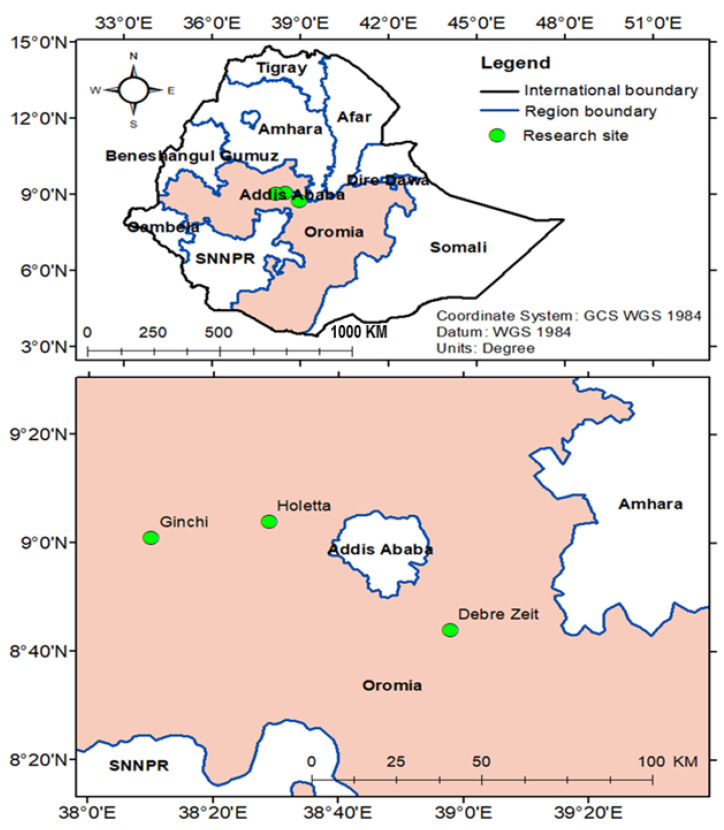
A map showing the locations of the three field experimental sites in Ethiopia.

Basically, three different noug materials were used in the present study: (i) twenty-eight landrace populations, (ii) ten “breeding populations”, and (iii) seven released cultivars (Table 1). The landrace populations were collected directly from farmers’ fields in four Federal States of Ethiopia (Amhara, Oromia, SNNPs and Tigray) during 2003. The altitude of origin of these samples (in meters above sea level) are given in Table 1. The seeds were rejuvenated in 2008 and 2012 by growing them in a greenhouse. The 10 breeding populations were developed through four generations of crossbreeding, selecting them based on their lipid content, oleic acid content, and/or seed size [27]. The seeds of the seven cultivars were obtained from Holeta Agricultural Research Center (HARC) of the Ethiopian Institute of Agricultural Research (EIAR). For the sake of simplicity, each population and cultivar will be referred to as a “genotype” from here on. In order to diminish the environmental effects due to differences in previous cultivations and to understand the interactions between genotypic and environmental variation, all the three materials of noug were grown in Ethiopia at three sites: Holeta (09°04′ N, 38°29′ E; 2400 masl), Ginchi (9°01′ N and 38°10′ E; 2300 masl), and Debrezeit (8°44′ N and 38°58′ E; 1900 masl) from June to December 2016 (Figure 1).

An incomplete block design within each site was used. Each genotype was planted on a 3 m by 2.1 m (6.3 sqm) plot in seven rows, with a 30 cm distance between rows. A distance of 1 m between the plots was used. A pollination net of 3.2 m (l) × 2.3 m (w) × 2 m (h) was used to cover the plants on each plot, just before flowering, to prevent gene flow between genotypes from different plots. Hand pollination was carried out between plants within each plot by gently rubbing mature flower heads (capitula) in the morning every other day for two weeks. Each genotype and plot was separately harvested at maturity and threshed, and clean seeds were used for laboratory analyses. The lipid content and fatty acid composition were evaluated separately for the seeds from each genotype, plot, and site. Due to resource limitations, 21 of the 28 landrace populations, nine of the 10 “breeding populations”, and six of the seven cultivars were used for protein and mineral content analyses.

### 2.2. Materials and Chemicals Used for the Determination of Nutrients in Noug Seeds

The reagents used for oil extraction, fatty acid derivatization, and gas chromatography (GC) analyses were supplied by Merck KGaA (Darmstadt, Germany) and were deemed suitable for these uses: acetic acid (glacial, 100%), methanol (anhydrous), chloroform (min 99.8%), sulphuric acid (95.0–98.0%), and hexane (≥95%). The methyl-heptadecanoate (≥99%) used as an internal standard for GC analysis of the oil was purchased from Sigma–Aldrich (Stockholm, Sweden). The nitric acid (BAKER INSTRA-ANALYZED^®^ reagent grade) used for digesting the fine powder of noug samples for mineral content analysis was supplied by Avantor™ (Radnor, PA, USA).

### 2.3. Lipid Extraction, Methylation of Fatty Acids, and Gas Chromatography (GC) Analysis

The content and composition of total seed oil and fatty acids were evaluated according to Bligh and Dyer [32], with the modifications described in Geleta et al. [27]. Thus, three replications, each having 10 seeds, from each genotype and site were homogenized using 1 mL 0.15 M acetic acid and 3.75 mL MeOH:CHCl_3_ (2:1, *v*/*v*), before adding 1.25 mL chloroform and 0.9 mL Millipore water, followed by vortexing. After centrifugation for 2 min at 3000 rpm, 50 mL of the chloroform phase of the extracted solution was transferred to a new glass tube, placed on a sand adjusted to 70 °C, and evaporated under a weak beam of liquid nitrogen. Following the complete evaporation of the chloroform solution, the fatty acids in the glass tube were methylated by adding 2 mL of 2% H_2_SO_4_ in methanol and incubating for 45 min at 90 °C. After the methylated solution was cooled down to room temperature, 200 nmol methyl-heptadecanoate (17:0-ME) was added to the solution as an internal standard, followed by the addition of 0.9 mL Millipore water and 2 mL hexane. The methylated solution was centrifuged for 2 min at 2000 rpm, and then 200 μL of the hexane phase containing the fatty acid methyl esters (FAMEs) was transferred to GC vials for analysis on Shimadzu GC model 17A (Kyoto, Japan), connected to a flame ionization detector [27]. The proportion of the fatty acids in the oil was calculated based on the relative percentage of the total peak area to that of the internal standard. The total lipid content of each analyzed seed sample was determined based on the total amount of the fatty acids (including the four major fatty acids; linoleic, oleic, palmitic, and stearic acids), as described in Geleta et al. [27]. This includes a calculation of the weight of each fatty acid and glycerol in the GC sample. Thereafter, the weight of triacylglycerol in the GC sample was calculated based on the weight of the fatty acids and the weight of glycerol in the sample. Then, the total lipid content was determined based on the weight of triacylglycerol and the dry weight of the seed sample [27].

### 2.4. Determination of the Protein Content

The total nitrogen content of each seed sample was determined on two sample replicates by dry combustion using an automated nitrogen analyzer (Model: Vario MAX CN, Elementar Americas) [33]. The protein content of each sample was then calculated from the estimated total nitrogen content (N) of the samples, using 5.6 [34] as the conversion factor.

### 2.5. Analysis of the Mineral Contents

A mineral content analysis of noug seeds was conducted on two sample replicates for each sample using a Perkin-Elmer Optima^®^ 8300 inductively coupled plasma optical emission spectrometer (ICP-OES, Perkin-Elmer, Waltham, MA, USA) [35,36]. Each sample was ground to a fine powder, and 0.5 g of each ground sample was diluted with water to a volume of 50 mL prior to analysis. The diluted samples were then placed in a microwave oven for digestion in a closed vessel with 7 mL HNO_3_ and 3 mL distilled water at a pressure and temperature of 375 psi and 185 °C, respectively. Blank solutions (without sample) were used as the control, and cross contaminations were checked. The mineral contents were recorded in micrograms of the mineral per gram of flour added to the solution (μg g^−1^) for each analyzed sample.

### 2.6. Statistical Analysis

An evaluation of the data was carried out using the statistical package, SAS version 9.4 (SAS Institute Inc., Cary, NC, USA). Due to the unbalanced dataset, the mixed model (PROC MIXED), followed by the Tukey-Kramer test, was used to calculate the effect of the genotypes and location, as well as their interaction on the analyzed components, such as the total lipid content, content of various fatty acids, and mineral and protein content. Principal component analysis was carried out to understand the genotypic relationship for the combined variation in total lipid, fatty acids, mineral, and protein contents.

## 3. Results and Discussion

### 3.1. Quantity and Quality of Lipids

The mean lipid content of the analyzed genotypes across the three environments ranged from 32.5% (*NG-85*) to 45.7% (*NG-99*) (Table 1), with about 15% of the genotypes showing a mean lipid content of 40% or above. Thus, the lipid content of the noug genotypes in the present study was in parity with the previously reported lipid content of 27–56% in this crop [19,23,24,25,26,27]. Previous studies have reported a two-fold variation between genotypes [27], with higher values in cultivated noug (41–43%) than in wild *Guizotia* (21–33%) [23] and similar levels in noug produced in the USA as in noug imported from India or Ethiopia to the USA (36–38%) [19]. Mixed model results revealed that the genotype and environment, as well as their interactions (G × E), influenced the lipid content in the evaluated noug genotypes (Table 2).

The fatty acid composition of the noug genotypes evaluated in the present study corresponded well with previous investigations, which found linoleic, oleic, palmitic, and stearic acids to be the major fatty acids in noug seeds [23,24,26,27]. Thus, linoleic acid was the predominant fatty acid in all analyzed samples, with values ranging from 72.3% (*NG-113*) to 77.8% (*NG-93*); the oleic acid concentration ranged from 5.2% (*NG-87*) to 9.2% (*NG-113*); and palmitic and stearic acids ranged from 7.8% (*NG-113*) to 9.6% (*NG-87*) and 7.2% (*NG-111*) to 10.0% (*NG-113*), respectively (Table 1). In previous studies, linoleic acid was found to range from 32–58% [26], 54–73% [23], and above 70% [24], while oleic acid was in the range of 6–11% [24], 5.4–27% [23], 3.3–31% [27], and 23–53% [26]. The present study showed clear effects of the environment and of the interactions between the genotype and environment on the content of fatty acids (Table 2). Such relationships between the cultivation environment and fatty acids composition have not been reported previously for noug. However, the genotype and environment, as well as their interactions, are well known to influence most components in plants [37,38], and the effects on the content of total lipids and fatty acids have been previously reported, e.g., in sunflower (*Helianthus annuus* L.), which is the closest crop to noug [39,40,41].

**Figure 2:**
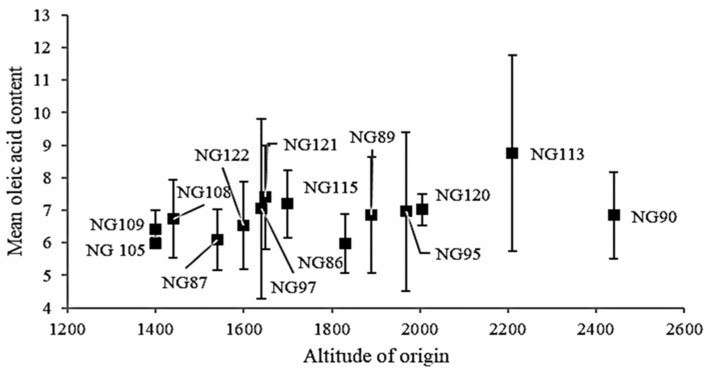
Variation of oleic acid content against altitude of genotype origin.

Among the fatty acids, the content of oleic acid was impacted by the environment and genotype x environmental interactions to a higher degree than the other fatty acids (Table 2). Additionally, previous studies have revealed a strong environmental impact on the oleic acid content in noug, where the altitudes of production have a significant effect on certain genotypes [27,42]. Specifically, noug genotypes with a high oleic acid content, which originate in low-altitude areas of Ethiopia, have been found to continuously produce high contents of oleic acid when grown at low altitudes, although the content of oleic acid was found to decrease substantially when grown at high altitudes [27]. The genotype, *NG-84*, has been reported in previous studies as such a high oleic acid genotype when grown at low altitudes, with a decreased oleic acid content at high altitudes and high oleic content (52%) when grown at 21 °C–25 °C under green-house conditions [27]. In the present study, this genotype did not produce such a high oleic acid content, although the levels were still higher at lower altitudes: a 9.8% oleic acid content at Debre Zeit (1900 masl), 7.1% at Ginchi (2300 masl), and 4.8% at Holeta (2400 masl). However, the present study also revealed a significantly positive (*p* < 0.01) Spearman rank correlation between high oleic acid values at low altitudes and the standard deviation of oleic acid content at various altitudes, independent of the altitude of origin of the samples (Figure 2). Thus, in this study, we were able to confirm a previous finding [27] that shows low-altitude areas of Ethiopia to be the origin for high-oleic-acid noug e.g., *NG-88* (referred to as *Tg-2* in Geleta et al. [27]), which produced 31.1% oleic acid under green-house conditions, collected at 1680 masl. This study also revealed that low-oleic-acid-content genotypes originated from low-altitude areas, showing less variation in the oleic acid content when grown at various altitudes (Figure 2).

Furthermore, a high content of oleic acid, when grown at high altitudes, was rarely found among the evaluated material. Therefore, our findings indicate limited opportunities to breed for and produce high levels of oleic acid in noug grown at high altitudes, while it would be worthwhile to focus on developing high-oleic-acid noug cultivars, specifically for production in lowland areas of Ethiopia and elsewhere. Opportunities to produce high-linoleic and oleic acid noug varieties would contribute an additional asset to the highly nutritious noug oil.

### 3.2. Protein Concentration in Noug Seeds

The protein concentration in the noug seeds used in this study varied between 17.8% and 30.0%, depending on the genotype and location of cultivation, and therefore corresponded with previous reports of a 25–28% protein concentration in noug seeds [43], although with a wider range of content. ANOVA showed no significant (at *p* < 0.05) variation in the seed protein concentration among genotypes (Table 3) or in the interactions between genotypes and locations, although a significantly (*p* < 0.05) higher seed protein concentration was noted when noug was grown in Ginchi (27.5%), compared with Holeta (25.4%; Table 4).

Most compounds, such as fatty acids, proteins, minerals, and phytochemicals in plant seeds are known to vary and be affected both by the genotype and the environment, and their interactions, and the magnitude of these effects on the content of the compounds depends on the genotypes and environments used [38,44]. In this study, the noug material was selected rather broadly to obtain genetic variation, and despite this, a limited genetic variation was found in the seed protein concentration, indicating little genetic variation in this trait for noug. Despite this, the cultivation environment had a significant effect on the trait. The effect of the environment on the grain/seed protein concentration is the result of complex processes, where both the protein accumulation and accumulation of starch and oil in the grain/seed influences the total protein concentration [45,46]. Thus, an increased protein concentration is often the result of a decrease in starch or oil in the seed/grain, which is often the result of stress conditions (heat, drought, insects, or pests), which negatively affect plant growth [38,47]. The fact that the plants were grown under rainfed conditions at both locations, and that Ginchi normally is a drier cultivation area than Holeta (the estimated average annual rainfall was 663 mm and 914 mm, respectively, as per the metrological records of the year), might be the explanation for the differences seen in the seed protein concentration. This implies that drought conditions for noug cultivation results in less dry matter, a lower yield, and a higher grain protein concentration.

### 3.3. Content of Mineral Elements

The mean seed mineral content of all the analyzed noug genotypes are presented in supplementary Table S1. Basically, the ANOVA analyses revealed a limited genotypic variation for all the minerals analyzed, with the exception of K (Table 3), while again the cultivation locations resulted in a significant variation for the majority of the minerals (Fe, K, Mg, Mn, S, Se, and Zn; Table 4). A higher mineral content was generally observed in the genotypes grown in Holeta, compared with Ginchi. However, the difference in mineral content between the two locations cannot be explained by a dilution effect of biomass variation, as the mineral content was not higher at Ginchi than at Holeta. Studies have shown that mineral content in cereal grains/seeds are known to be genetically determined but also highly dependent on the variation in the soil mineral content [38,48]. Previous studies on the mineral content of a range of grains/seeds have shown a wide variation in mineral content between types of grains/seeds and also between genotypes [30,36,48]. The fact that a limited variation was found between noug genotypes in mineral composition in the present study needs to be further evaluated. Here, we assume that the variation in soil mineral content of the two locations is the major determinant of the seed mineral content of noug. Thus, the differences in seed mineral content between the noug genotypes grown at the two locations was most likely caused by differences in the mineral content between the two localities. Therefore, environments with suitable soil conditions should be considered for noug production, if an increased seed mineral content is desired.

### 3.4. Variation in Oil Content, Fatty Acids, Seed Protein Concentration, and Minerals across Genotypes and Locations

**Figure 3:**
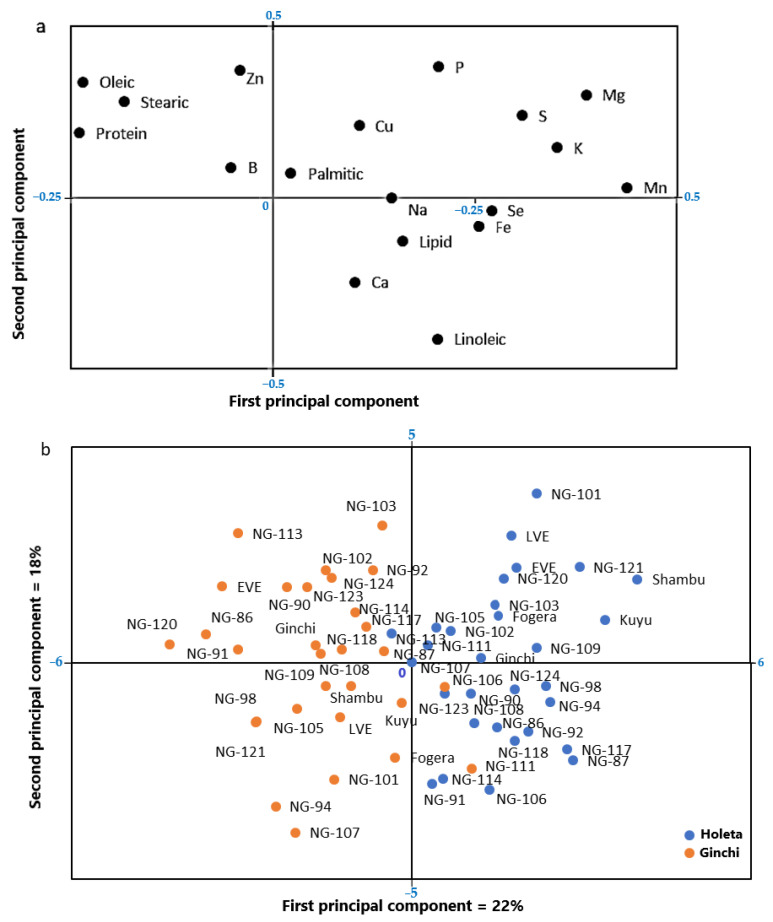
Loading (**a**) and score (**b**) plots from the principal component analysis of the total lipid, fatty acid, seed protein, and mineral contents of noug genotypes grown at two locations (Holeta and Ginchi).

Principal component analysis, used to depict the variation of all evaluated variables (total lipid content, fatty acid composition, seed protein concentration, and minerals) (Figure 3a), clearly revealed the effect of the growing locations on the levels of these variables (Figure 3b).

Basically, all genotypes grown at Ginchi showed negative values for the first principal component, while the same genotypes grown at Holeta showed positive values (Figure 3b), corresponding with the abovementioned findings of the importance of the growing locations for the content and composition of the evaluated variables. The first principal component explained 22% of the variation, while 18% of the variation was explained by the second principal component. The seed protein concentration showed a negative value for the first principal component, while most of the minerals showed positive values (Figure 3a), thus verifying the higher seed protein concentration in the samples from Ginchi and higher values for the mineral content in the samples from Holeta, as also described above through the analyses of variance and mean value comparisons. No clear genotypic differences in the protein and mineral contents were shown in the principal component analysis (compare Figure 3a,b). As for the lipid and fatty acid contents, the principal component analysis revealed the interacting effects of the genotypes and locations of cultivation, which might warrant further analyses of the stability of the contents.

### 3.5. Nutritional Value of Noug in Comparison with Other Oilseed Crops

The nutritional quality of noug can be divided into two parts: (i) the quality of noug oil production, which is principally determined by the total lipid content and composition of the fatty acids; and (ii) the quality of the whole seed/cake for human nutrition, which is basically determined by the fatty acid, protein, and mineral composition. The total lipid content (of importance for vegetable oil production) in noug had a close parity with that found in a range of other representative oilseed crops, all of which have recently been described in a monograph of important oil crops [49,50,51,52,53]. Higher values than in noug have been reported for sesame, groundnut, and castor bean, while lower values have been reported for soybean and cottonseed (Table 5).

Considering the total lipid content, noug has the potential to develop into an oil crop of great importance. However, the nutritive value and quality of oilseeds is not only dependent on the total lipid content, but even more so on the fatty acid composition of the seed oil [72]. The fatty acid composition is also of nutritional relevance if the oil crop is used as whole seed for human consumption. Here, unsaturated fatty acids are of importance, as these are hypocholesterolemic, contributing positive health effects to the blood serum cholesterol. In this respect, linoleic and oleic acids are among unsaturated fatty acids with hypocholesterolemic properties in noug and many other oil crops, contributing positively to health when consumed [72,73,74]. Of specific interest here is the fact that linoleic acid is polyunsaturated and an essential fatty acid, as it cannot be produced by the human body but needs to be included in the diet [74]. The comparison of the fatty acid composition in noug with that of other oilseed crops (Table 5) revealed that noug has high levels of linoleic acid (72–78%), with only safflower showing a comparable content (74–78%). However, the content of oleic acid was generally low in noug (5–8%), and only castor bean has similar low values (3–5%), although the major fatty acid in castor bean is ricinoleic acid [75]. Despite this, the high linoleic acid content in noug may place it among the high-quality oil crops. Of relevance in this context is the storage conditions of the noug oil, which should be in airtight containers to prolong the shelf-life and to minimize the oxidation of the polyunsaturated fatty acids. Palmitic and stearic acids, also found in noug and many other oil crops, are saturated fatty acids with a hypercholesterolemic or neutral effect on the blood serum cholesterol [73]. The present comparison showed that noug has a similar level of palmitic acid as the majority of the other oilseeds (7–10%; Table 5), but with a relatively high content of stearic acid (7–10% in relation to 1–6% in most of the other oil crops). However, the high total lipid content, combined with the high content of linolenic acid, makes noug nutritionally unique for vegetable oil production among the oilseed crops.

The protein concentration and composition are of importance if the oil crops should be used for human consumption as whole seed or seed cake. Additionally, when it comes to the seed protein concentration, noug is similar to the other oil crops. As can be seen in Table 5, only soybean and cottonseed have a higher seed protein concentration than noug, while sesame and linseed are in parity, and sunflower, safflower, and Brassica all showed lower values. The present study did not evaluate the amino acid composition of noug seed protein. Previous studies [76] have indicated that the amino acid composition of noug seeds is fairly balanced for human consumption. Protein isolates prepared from noug have shown a high in vitro protein digestibility, as well as a high iron and zinc dialysability [43]

Similarly, as for the seed protein concentration, the mineral composition is of great importance when the whole seed or cake of the oilseed crops are used for human nutrition. For mineral composition, the content of Fe in noug seeds showed a fairly high variation in the present study (Table 5). The high Fe-containing noug seeds were in parity with the high Fe content previously reported for cotton seed and castor bean [53,71]. However, the content of Zn in noug was on the lower end (2.1 to 5.1 mg 100 g^−1^), as compared to many of the other seeds of the oilseed crops (Table 5). However, lower levels of Zn have been reported for Safflower and Mustard (Table 5) [59,63]. A sufficient intake of Fe and Zn is of relevance in most parts of the world [37], including Ethiopia. However, 10.5% to 29.6% of breastfeeding mothers in different rural areas in Ethiopia were found to have an Fe deficiency [77,78]. Furthermore, Zn deficiency, which results in stunting among children aged between 6 and 35 months was reported in both rural and urban areas in Ethiopia, as a result of a low dietary intake of Zn in the country’s population in general, also including breastfeeding mothers [79]. If noug seed cake, whole-seeds, or flour should be increasingly used as a food source in Ethiopia and elsewhere, the increasing mineral content should be considered as a major breeding goal for the crop to contribute to increasing human wellness.

## 4. Conclusions

Noug is unique among oil crops in having a high total seed lipid content, with a high proportion of linoleic acid, contributing to the health-promoting properties of its vegetable oil. For the use of its seed cake, whole-seed, or seed meal, the fatty acid composition, with a high content of linoleic acid content, is beneficial, and noug also has a high seed protein concentration and a high Fe content, contributing to its nutritional potential. Unfortunately, the Zn content and oleic acid content are on the lower end of that found in other oilseed crops, partly hampering the quality of both the vegetable oil and whole seed/cake of noug when used for human consumption. Noug genotypes with a high oleic acid content are available when grown at low altitudes in Ethiopia, making them suitable to breed for high-oleic-acid-content noug varieties for these growing environments. However, when these genotypes are grown at high altitudes, the oleic acid content is reduced. Thus, a lack of opportunities to grow high oleic acid genotypes at high altitudes seems to prevail. The environmental conditions are also of higher importance than the genotypes in determining the grain protein concentration and mineral content in the seeds, and both factors are of importance if the oil crops are to be used as whole seed/cake for human consumption. Thus, for a high seed protein concentration, dry conditions, reducing the yield, are needed, which is not a suitable solution, while the soil mineral content is a major factor determining the mineral content of noug seeds. Despite the minor influence of the genotypes on the quality attributes, novel genomic tools, such as the recently described transcriptome-based SNP markers and the developed KASP markers, e.g., in [80], might be of importance for the future development of noug with improved nutritional properties.

## Figures and Tables

**Table 1:** Least square means (%), obtained from the mixed model ± standard error, of the total oil content and content of the fatty acids of noug genotypes and the altitude of origin of the various samples.

Genotype	Total Lipid Content	Linoleic Acid	Oleic Acid	Palmitic Acid	Stearic Acid	Altitude of Origin (Masl)
NG-83 ^b^	39.8 ± 1.6	75.4 ± 1.0	6.4 ± 0.6	9.5 ± 0.3	7.7 ± 0.5	na
NG-84 ^b^	41.2 ± 1.6	73.2 ± 1.0	8.3 ± 0.6	8.6 ± 0.3	8.1 ± 0.5	na
NG-85 ^a^	32.5 ± 2.7	76.9 ± 1.0	6.7 ± 1.1	8.6 ± 0.5	7.4 ± 0.9	1740
NG-86 ^a^	38.1 ± 1.6	74.0 ± 1.0	6.6 ± 0.6	9.5 ± 0.3	9.5 ± 0.5	1830
NG-87 ^a^	36.0 ± 1.6	75.4 ± 1.0	5.2 ± 0.6	9.6 ± 0.3	9.3 ± 0.5	1540
NG-88 ^a^	35.5 ± 2.7	77.2 ± 1.7	6.2 ± 1.1	8.5 ± 0.5	7.6 ± 0.9	1680
NG-89 ^a^	38.1 ± 1.6	75.3 ± 1.0	7.1 ± 0.6	9.0 ± 0.3	8.8 ± 0.5	1890
NG-90 ^a^	38.7 ± 1.6	75.7 ± 1.0	6.9 ± 0.6	8.5 ± 0.3	8.5 ± 0.5	2440
NG-91 ^a^	38.5 ± 1.9	76.0 ± 1.2	7.4 ± 0.8	8.4 ± 0.4	7.6 ± 0.6	1890
NG-92 ^b^	44.4 ± 1.9	76.5 ± 1.2	6.9 ± 0.8	8.6 ± 0.4	7.6 ± 0.6	1860
NG-93 ^a^	36.4 ± 1.9	77.8 ± 1.2	5.5 ± 0.8	8.0 ± 0.4	8.1 ± 0.6	2425
NG-94 ^a^	34.5 ± 1.9	77.2 ± 1.2	5.9 ± 0.8	8.6 ± 0.4	8.3 ± 0.6	1920
NG-95 ^a^	39.0 ± 1.6	74.8 ± 1.0	7.6 ± 0.6	8.3 ± 0.3	9.0 ± 0.5	1968
NG-96 ^a^	36.8 ± 2.7	77.3 ± 1.7	6.2 ± 1.1	8.6 ± 0.5	7.3 ± 0.9	1820
NG-97 ^a^	39.3 ± 1.6	77.2 ± 1.0	6.6 ± 0.6	8.7 ± 0.3	7.4 ± 0.5	1640
NG-98 ^a^	39.2 ± 1.6	75.4 ± 1.0	7.0 ± 0.6	8.4 ± 0.3	8.5 ± 0.5	1590
NG-99 ^b^	45.7 ± 1.9	76.4 ± 1.0	7.1 ± 0.8	8.3 ± 0.4	8.5 ± 0.6	2460
NG-101 ^b^	40.0 ± 1.9	75.4 ± 1.2	6.8 ± 0.8	8.9 ± 0.4	8.5 ± 0.6	2400
NG-102 ^a^	37.0 ± 1.9	74.7 ± 1.2	7.7 ± 0.8	8.7 ± 0.4	8.2 ± 0.6	2430
NG-103 ^a^	35.4 ± 1.9	75.2 ± 1.2	7.0 ± 0.8	8.5 ± 0.4	9.2 ± 0.6	2045
NG-105 ^a^	34.9 ± 1.6	76.4 ± 0.2	6.4 ± 0.6	8.3 ± 0.3	8.3 ± 0.5	1400
NG-106 ^a^	37.8 ± 1.6	76.3 ± 1.0	7.3 ± 0.6	8.5 ± 0.3	8.3 ± 0.5	1840
NG-107 ^b^	40.7 ± 1.9	76.3 ± 1.2	6.5 ± 0.8	7.9 ± 0.4	8.8 ± 0.6	1945
NG-108 ^a^	33.6 ± 1.6	75.5 ± 1.0	6.9 ± 0.6	9.0 ± 0.3	8.3 ± 0.5	1440
NG-109 ^a^	33.1 ± 1.6	74.9 ± 1.0	6.8 ± 0.6	9.0 ± 0.3	8.8 ± 0.5	1400
NG-111 ^b^	39.4 ± 1.9	75.8 ± 1.2	7.4 ± 0.8	8.8 ± 0.4	7.2 ± 0.6	2590
NG-112 ^a^	35.7 ± 1.9	76.4 ± 1.2	6.4 ± 0.8	8.3 ± 0.4	8.2 ± 0.6	2155
NG-113 ^a^	37.2 ± 1.6	72.3 ± 1.0	9.2 ± 0.6	7.8 ± 0.3	10.0 ± 0.5	2210
NG-114 ^b^	35.6 ± 1.6	75.2 ± 1.0	7.1 ± 0.6	9.0 ± 0.3	8.5 ± 0.5	na
NG-115 ^a^	41.4 ± 1.6	77.2 ± 1.0	7.1 ± 0.6	8.4 ± 0.3	7.8 ± 0.5	1700
NG-117 ^b^	43.7 ± 1.9	76.6 ± 1.2	6.1 ± 0.8	8.8 ± 0.4	8.3 ± 0.6	2210
NG-118 ^a^	39.2 ± 1.9	76.2 ± 1.2	6.6 ± 0.8	8.5 ± 0.4	8.1 ± 0.6	2540
NG-119 ^a^	35.5 ± 2.7	74.5 ± 1.2	7.5 ± 1.1	8.8 ± 0.5	9.3 ± 0.9	2370
NG-120 ^a^	39.2 ± 1.6	75.5 ± 1.0	6.7 ± 0.6	8.6 ± 0.3	8.9 ± 0.5	2005
NG-121 ^a^	37.8 ± 1.6	75.3 ± 1.0	7.0 ± 0.6	8.4 ± 0.3	8.8 ± 0.5	1650
NG-122 ^a^	32.8 ± 2.7	75.9 ± 1.7	6.3 ± 1.1	8.8 ± 0.5	8.9 ± 0.9	1790
NG-123 ^a^	38.1 ± 1.6	75.7 ± 1.0	6.9 ± 0.6	8.5 ± 0.3	8.5 ± 0.5	1600
NG-124 ^b^	39.1 ± 1.6	75.9 ± 1.0	6.4 ± 0.6	8.7 ± 0.3	8.1 ± 0.5	na
EST ^c^	34.5 ± 2.7	73.5 ± 1.7	8.2 ± 1.1	8.6 ± 0.5	9.8 ± 0.9	na
EVE ^c^	35.9 ± 1.6	75.2 ± 1.0	7.5 ± 0.6	8.7 ± 0.3	8.6 ± 0.5	na
FOG ^c^	34.3 ± 1.6	77.4 ± 1.0	5.8 ± 0.6	8.5 ± 0.3	7.7 ± 0.5	na
GIN ^c^	37.4 ± 1.6	75.4 ± 1.0	6.3 ± 0.6	9.0 ± 0.3	8.8 ± 0.5	na
KUY ^c^	37.6 ± 1.6	76.5 ± 1.0	6.6 ± 0.6	8.6 ± 0.3	8.0 ± 0.5	na
LVE ^c^	33.0 ± 1.6	76.0 ± 1.0	6.2 ± 0.6	8.4 ± 0.3	8.9 ± 0.5	na
SHA ^c^	34.6 ± 1.6	76.3 ± 1.0	6.2 ± 0.6	8.5 ± 0.3	8.3 ± 0.5	na

^a^ landrace population, ^b^ breeding population, ^c^ released cultivar. na = not applicable.

**Table 2:** F-values for the sources of variation (i.e., environments (E), genotype (G), and G × E interactions) from the mixed model for the total oil content and fatty acids in noug.

Source of Variation	DF ^†^	Total Lipid Content	Linoleic Acid	Oleic Acid	Palmitic Acid	Stearic Acid
Environment (E)	2	29.6 ***	32.7 ***	38.7 ***	ns	20.5 ***
Genotype (G)	44	2.7 ***	ns	ns	ns	ns
G × E	44	2.3 ***	1.4 ***	4.0 ***	1.4 *	4.1 ***

^†^ DF = degrees of freedom, * and *** = Significant at *p* < 0.05 and 0.005, respectively, ns = not significant at *p* < 0.05.

**Table 3:** Mean squares from the analyses of variance (ANOVA) of the seed protein concentration and content of minerals in noug.

Source of Variation	DF ^†^	Protein (10^1^)	Minerals											
			B (10^1^)	Ca (10^5^)	Cu (10^1^)	Fe (10^5^)	K (10^7^)	Mg (10^6^)	Mn (10^4^)	Na (10^2^)	P (10^6^)	S (10^6^)	Se (10^−3^)	Zn (10^2^)
Location	1	8.2 *	0.6	0.7	0.1	4.9 *	2.1 ***	5.1 **	1.5 ***	0.4	2.3	1.2 ***	4.9 ***	2.1 *
Genotype	35	0.6	1.3	1.9	4.2	0.9	0.1 **	0.6	0.00	0.7	0.8	0.0	0.2	0.2
Error	35	0.4	1.6	1.5	3.6	0.9	0.0	0.6	0.00	1.4	1.3	0.0	0.2	0.4

^†^ DF = degrees of freedom, *, ** and *** = Significant at *p* < 0.05, 0.01 and 0.005, respectively.

**Table 4:** Mean values of the seed protein concentration (%) and of mineral content (µg g^−1^) for those minerals showing a significant difference between the two experimental locations.

Location	N	Protein	Minerals						
			Fe	K	Mg	Mn	S	Se	Zn
Holeta	36	25.4 b	347 a	9254 a	4080 a	46.8 a	3253 a	0.043 a	45.0 b
Ginchi	36	27.5 a	181 b	8170 b	3547 b	18.0 b	3000 b	0.027 b	48.4 a

Numbers within a column followed by the same letter do not differ significantly (*p* < 0.05).

**Table 5:** Content of total lipids, fatty acids, protein, Fe, and Zn in major oilseed crops seeds.

Oilseeds		Total Lipid Content (%)	Fatty Acids (%)				Protein mg/g	Fe mg/100 g	Zn mg/100 g
Common Name	Scientific Name		Linoleic Acid	Oleic Acid	Palmitic Acid	Stearic Acid		(10^1^)	
Noug	*Guizotia* *abyssinica*	32.5–45.7 ^a^	72.2–77.8 ^a^	5.2–8.3 ^a^	7.8–9.6 ^a^	7.2–10.0 ^a^	24.4–27.5 ^a^	1.2–12.5 ^a^	2.0–5.3 ^a^
Sunflower	*Helianthus* *annuus*	20.5–23.9 [54]	32.2–54.3 [54]	31.9–56.9 [54]	6.6–6.8 [54]	4.0–4.1 [54]	10.0–27.1 [55]	0.5–0.7 [49]	5.0–7.6 [49]
Sesame	*Sesamum* *indicum*	49.5–51.3 [25]	41.0–45.0 [25]	39.5–43.0 [25]	8.4–10.3 [25]	4.5–5.8 [25]	23.1–25.2 [56]	9.3 [50]	3.8 [50]
Safflower	*Carthamus* *tinctorium*	36.0–41.0 [57]	74.6–78.2 [57]	11.2–14.2 [57]	6.0–6.7 [57]	2.0–2.6 [57]	17.6–18.1 [58]	3.5–4.0 [59]	1.5–2.1 [59]
Groundnut	*Arachis* *hypogae*	44.4–47.6 [60]	28.3–37.8 [60]	42.7–53.1 [60]	8.4–12.5 [60]	1.9–3.9 [60]	25.8 [61]	2.3 [51]	3.3 [51]
Mustard	*Brassica* *carinata*	39.8–46.4 [28]	17.3–19.9 [28]	10.6–12.1 [28]	3.0–3.7 [28]	1.4–2.3 [28]	32.4–36.4 [62]	1.3 [63]	0.07 [63]
Flaxseed	*Linum* *usitatissimum*	30.0–45.8 [25]	10.0–17.4 [25]	11.3–29.4 [25]	4.3–12.3 [25]	1.9–6.3 [25]	20.0–30.0 [64]	2.7 [52]	4.0 [52]
Soybean	*Glycine* *max*	20.0–22.0 [65]	50.0–60.0 [65]	22.0–25.0 [65]	7.0–10.0 [65]	2.0–5.0 [65]	37.3–40.6 [66]	7.1–8.2 [67]	4.2–11.7 [67]
Cottonseed	*Gossypium* spp.	17.5–27.0 [25]	50.5–55.0 [25]	20.0–25.0 [25]	2.0–2.5 [25]	2.5–3.3 [25]	34.2–46.3 [68]	12.0 [53]	6.1 [53]
Castor bean	*Ricinus* *communsis*	61.6–72.3 [69]	3.5–4.5 [69]	2.9–3.6 [69]	1.1–1.3 [69]	0.9–1.2 [69]	21–48 [70]	17.0 [71]	13.0 [71]

^a^ = current study.

## Data Availability

The research data presented in this study are available within the manuscript and in the supplementary material, Table S1. The raw data can be obtained by contacting the corresponding author.

## Supplementary Materials

The following are available online at https://www.mdpi.com/article/10.3390/foods10081778/s1. Table S1: Mean values with standard deviations of the mineral content for the evaluated genotypes.
