# Supplementary material for: Nutritional Profile of the Ethiopian Oilseed Crop Noug (Guizotia abyssinica Cass.): Opportunities for Its Improvement as a Source for Human Nutrition
Source: Foods. 2021 Jul 31;10(8):1778. doi: 10.3390/foods10081778 (PMC8393925; doi:10.3390/foods10081778)
Supplement: Supplementary file 1 [file foods-10-01778-s001.zip › foods-1315121-supplementary.pdf]

Table S1. Mean mineral (μgg^{-1}) and total nitrogen (mgg^{-1}) contents of 36 noug genotypes grown at two locations (Ginchi and Holeta) in Ethiopia

Genotype	B	Ca	Cu	Fe	K	Mg	Mn	Na	P	S	Se	Zn	TN
NG-84[b]	18	3839	18	350	7795	3792	42	45	9002	3099	0.022	45	46.1
NG-86[a]	18	3553	21	145	8154	3496	27	33	7812	3084	0.029	44	41.3
NG-87[a]	17	3744	19	368	8778	3652	41	28	9333	3269	0.027	46	43.4
NG-90[a]	19	3160	18	126	8481	3885	28	22	8950	3257	0.033	47	46.0
NG-91[a]	17	3542	17	179	7970	3477	25	19	8135	3133	0.018	44	44.9
NG-92[b]	17	3253	30	158	8383	4013	29	29	9259	3178	0.031	51	44.6
NG-93[a]	23	4036	36	1250	8524	3598	41	38	8432	2867	0.030	52	39.0
NG-94[a]	21	4602	20	164	8776	3480	26	22	8660	3077	0.034	43	42.8
NG-95[a]	24	3978	16	739	7096	3303	31	34	8766	2964	0.037	46	45.8
NG-97[a]	29	4116	22	358	8100	3592	38	28	8219	2997	0.031	49	39.3
NG-98[a]	22	3865	14	168	8955	3554	28	25	8943	3185	0.036	44	45.0
NG-99[b]	22	3939	16	203	9346	6849	25	29	9364	3113	0.034	51	46.0
NG-101[b]	21	3780	18	171	9840	3897	30	26	9113	3160	0.035	52	43.6
NG-102[a]	20	3691	18	167	9522	3750	29	25	9341	3267	0.027	53	45.7
NG-103[a]	21	3638	19	241	9805	4322	34	28	10589	3269	0.026	51	48.6
NG-105[a]	22	4263	16	159	8490	3569	34	30	8297	3313	0.035	46	48.6
NG-106[a]	19	4222	16	329	8467	3641	36	28	9113	3137	0.040	46	42.7
NG-107[b]	19	4311	15	221	9002	3226	29	26	8715	2927	0.017	41	46.9
NG-108[a]	20	3871	15	120	8042	3664	33	35	9238	3218	0.041	43	46.0
NG-109[a]	19	3335	15	406	8186	3692	40	26	9222	3302	0.036	45	44.0
NG-111[b]	18	3774	18	375	7988	3607	32	36	8913	3115	0.043	48	41.5
NG-112[a]	17	3349	15	123	9842	3748	36	35	9986	3370	0.083	44	46.9
NG-113[a]	18	3384	15	122	8007	3768	32	23	9988	3134	0.033	46	45.4
NG-114[b]	17	3642	15	127	7902	3815	31	22	9533	3152	0.032	47	46.7
NG-117[b]	17	3703	21	275	9257	3633	32	33	9503	3076	0.036	50	45.1
NG-118[a]	17	3677	16	127	8809	3554	28	26	9108	3304	0.044	46	44.3
NG-120[a]	20	3460	18	158	8513	3542	35	31	9667	3009	0.033	45	48.8
NG-121[a]	20	3718	25	320	8620	3671	35	44	9345	2968	0.031	47	41.7
NG-123[a]	20	3409	25	144	7914	3782	29	30	9468	3066	0.032	48	46.5
NG-124[b]	19	3480	23	160	8187	4031	30	31	10399	2989	0.031	52	43.5
EVE[c]	19	3894	18	153	9323	3953	32	26	9820	3135	0.031	46	46.5
FOG[c]	21	3752	21	287	9093	3908	29	35	9688	2980	0.033	45	43.3
GIN[c]	19	3753	25	195	9036	3651	33	26	9052	2966	0.032	46	43.8
KUY[c]	18	3537	18	156	9845	4090	37	34	9603	3266	0.053	42	44.4
LVE[c]	20	3813	22	320	9598	3862	35	40	9653	3087	0.027	46	46.5
SHA[c]	22	3782	18	432	9978	4231	37	37	10412	3126	0.033	46	47.4

[a] landrace population, [b] breeding population, [c] released cultivar. TN = Total nitrogen
